# “Although I’m mentally ill, that doesn’t mean that I’m not also physically ill” – barriers, facilitators and diagnostic overshadowing in healthcare for individuals with lived experience of mental ill-health

**DOI:** 10.3389/fpubh.2026.1739409

**Published:** 2026-03-31

**Authors:** Katrin Schäfer, Hanna M. Mües, Magdalena Agnieszka Wrzesińska, Magdalena Kostyła, Jarosław Rakoczy, Rosa Gomez Trenado, Cristina Fernández García, Marta Pisano González, Noelia Mancebo Salas, Thanos Gogos, Ioanna Zioga, Vasiliki Radaios, Alejandro Gil-Salmeron, Igor Grabovac

**Affiliations:** 1 Department of Social and Preventive Medicine, Center for Public Health, Medical University of Vienna, Vienna, Austria; 2 Department of Psychosocial Rehabilitation, Medical University of Lodz, Lodz, Poland; 3 Area of Social Work Madrid Health Service (SERMAS), Faculty of Social Work Complutense, University of Madrid, Madrid, Spain; 4 Regional Health Service of the Principality of Asturias (SESPA), Health Research Institute of the Principality of Asturias (ISPA), Asturias, Spain; 5 Ministry of Health of the Principality of Asturias (CSPA), Health Research Institute of the Principality of Asturias (ISPA), Asturias, Spain; 6 Government of the Community of Madrid, Madrid, Spain; 7 University Mental Health, Neurosciences and Precision Medicine Research Institute “COSTAS STEFANIS” (UMHRI), Athens, Greece; 8 PROLEPSIS Civil Law Non-Profit Organization of Preventive Environmental and Occupational Medicine, Athens, Greece; 9 International Foundation for Integrated Care, Oxford, United Kingdom; 10 Comprehensive Cancer Centre, Medical University of Vienna and Vienna General Hospital, Vienna, Austria

**Keywords:** co-creation, diagnostic overshadowing, health equity, lived experience, patient navigation

## Abstract

**Background:**

People with lived experience of mental ill-health (PWLE) face inequities in health care, including experiences of stigma and discrimination due to mental ill-health, as well as diagnostic overshadowing. This study aimed to investigate facilitators, barriers, and experiences of diagnostic overshadowing of PWLE within four European health care systems (Austria, Greece, Poland, and Spain).

**Methods:**

This qualitative interview study included PWLE and other relevant stakeholders (*N* = 85) with an age range from 22 to 76 years (*M* = 47.01, *SD* = 12.95). Semi-structured interviews were transcribed verbatim and thematically analysed.

**Results:**

The analyses revealed three themes: (1) “Factors located within the health care system,” (2) “Factors in regard to the personal situation,” and (3) “Impact of diagnostic overshadowing on health.” The results indicated that experiences of not being taken seriously and issues of stigma and discrimination in the health care setting were recognized across all countries. Additionally, self-stigmatization was mentioned as an additional burden.

**Conclusion:**

These findings show the limitations in access to and utilization of health care services by PWLE across different health care systems. Additional efforts are needed to improve access and utilization of physical health care for PWLE to promote equity in health care and, ultimately, health.

## 1 Introduction

Mental and physical ill-health are closely related. However, people with lived experience of mental ill-health (PWLE)—individuals who themselves have past or present experiences with mental ill-health, which might differ in severity over the life course—often face shortcomings in prevention and health promotion services, particularly regarding non-communicable diseases (1–3). A systematic review and meta-analysis by Chan and colleagues (4) found a shorter life expectancy of more than 15 years in people with severe mental health disorders such as eating disorders, schizophrenia spectrum disorders, and personality disorders, with the greatest reduction of more than 20 years in the case of substance use disorders. These discrepancies can be attributed to various factors beyond mental health problems, including health care access and utilization [e.g., higher mortality after myocardial infarction and less statin use in diabetes treatment (1)], health-related behaviors [e.g., tobacco use (5)], and preventive efforts [e.g., later or unplanned diagnosis of cancer (2)]. Preventive measures to address risk factors for non-communicable diseases can be challenging at the individual level, and the likelihood of their utilization is lower in PWLE compared with individuals without mental ill-health (6). The interplay of mental and physical health issues can influence health care utilization, health-related behaviors, and disease prevention, resulting in an excess burden of disease, especially when it comes to highly preventable diseases (7–9).

Barriers to accessing and utilizing preventive measures, including experiences of stigmatization in healthcare settings, contribute to disparities that further perpetuate inequity in prevention at personal, societal, and systemic levels. In a systematic review on schizophrenia stigmatization among mental health professionals, differences in stereotype beliefs between different professions were reported (10). Among all groups of mental health professionals, medical doctors reported the least empathy, and psychiatrists showed the most desire for social distance (10). Four types of stigmata experienced by PWLE were mentioned in a recent Lancet commission report: (1) *self-stigma* or internalized stigma, (2) *stigma by association*, (3) *public and interpersonal stigma*, and (4) *structural (systemic or institutional) stigma* (11). Particular attention should be paid to a concept closely related to stigma in health care settings: *diagnostic overshadowing* (DO). DO, which emerged in the 1980s, is often described as an *“evolutionary concept*” (12) as it lacks a widely accepted final definition; however, it generally refers to “a process by which physical symptoms are misattributed to mental illness*”* (13). DO can include a variety of biases, such as *anchoring bias* (reliance upon initial impressions even after receiving additional, deviating information), *premature closure bias* (ceasing investigation after the formulation of an initial diagnosis), or *implicit bias* (preconceptions of race, ethnicity, gender, diagnoses, etc., and their effects on patient interaction and care) (12). Thus, it is important to notice that DO is different from stigma as it especially occurs within clinical decision making (14). While stigma is a primary contributor to DO, it is important to distinguish the two concepts: DO refers specifically to processes that occur within clinical decision-making, whereas stigma represents a broader social devaluation and discrimination. Beyond stigma, structural factors of healthcare systems, as well as limited knowledge, skills, and education of health professionals, may also contribute to DO (14).

To counteract DO in health care settings, facilitators for access to health care utilization for PWLE should be recognized. Facilitators described by PWLE include the need for more empowerment and concrete support to change health-related behaviors (15). To consider the individual realities of life and the associated needs, including challenging symptoms of mental health disorders (e.g., changes in sleep and circadian rhythm), a person-centred approach and tailoring processes to individual needs is recommended (14). In a clinical study using a randomized controlled trial design, PWLE receiving a tailored intervention were more likely to quit smoking after six months than controls with regular service use (16). Additionally, modifiable risk factors such as tobacco smoking, unhealthy diet, or lack of physical activity are often shared between chronic diseases. Interventions targeting said risk factors have the potential to reduce the risk for multiple diseases at once.

Moreover, PWLE are confronted with the reality of mental health symptoms, which can be burdensome, as they may interfere with efforts in health-promoting behaviors and could be a barrier while utilizing the health care system, in addition to the potential occurrence of DO. This underscores the need for more empowerment and support (15) for PWLE in sustaining and promoting their own health. Prevention is not only the first step to reduce the risk for non-communicable diseases, but it is also cost-saving, not only for individuals (e.g., the financial burden of tobacco use) but on a systemic level as well. The literature shows discrepancies in health and preventive care, and the call for health equity for PWLE has become apparent in the reduced quality of health care (11, 15) and lower life expectancy (4, 17). To bridge this gap, the lived realities of PWLE need to be considered. This requires research to actively engage with the experiences and views of PWLE and other relevant stakeholders (e.g., caregivers) to develop a comprehensive understanding of the current situation and the specific needs of PWLE.

Finally, the objective of this qualitative study was to assess the barriers and facilitators PWLE face within four European health care settings (Austria, Greece, Spain, and Poland) in order to explore the experiences of PWLE regarding physical health care in a European context and to gain deeper insights into the health-related inequities encountered by PWLE. Given the systemic inequities of PWLE in terms of physical health care in general and prevention specifically, the aims of this study are twofold. Firstly, the barriers and facilitators in access to health care and preventive measures for individuals with mental ill-health need to be investigated. Secondly, the contribution of diagnostic overshadowing to said barriers and their manifestation in the experience of PWLE needs to be outlined.

## 2 Materials and methods

### 2.1 Study design

This study is part of the CO-CAPTAIN project, which focuses on primary cancer prevention in PWLE and is funded by the European Union’s Horizon Europe Programme. It is situated in a broader scope of a co-creation approach, with representatives of PWLE as part of the project consortium. The four countries included in the study (Austria, Greece, Poland, and Spain) were determined by the CO-CAPTAIN consortium structure and represent the implementation sites of the project.

In this study, a qualitative approach was adopted using semi-structured qualitative single-person interviews. Interview guidelines were developed for this study, reviewed by one PWLE (male, 41 years old, Austrian citizen, experienced in co-designed research), and amended where necessary. Additionally, to review, the guidelines have been tested with one PWLE. Co-creation of the interview guidelines included changes in terms and language, for example, a stronger focus on ‘experiencing/having mental health problems’ than ‘being mentally ill’. Feedback loops continued until the PWLE approved the guidelines. The guidelines were translated into the languages of each pilot site (German, Greek, Spanish, and Polish). Although the analyses in this report do focus on certain aspects, all of the domains covered in the interview guidelines are outlined for transparency: Sociodemographic data; Perceived physical health and cancer risk factors in people with mental health problems; Relevance, knowledge, and use of cancer prevention measures; Barriers and facilitators on the individual, institutional/organisational and systemic level to the access and use of the health care system; Perception of and input on implementing a Patient Navigation Model for cancer prevention in people with mental health problems; Opinions on the utilization of an App as part of the implementation phase of the Patient Navigation Model. The English version of the interview guidelines is available in the Supplementary file 1. This study also aims to promote participatory activities in research while making efforts to avoid patronizing actions.

### 2.2 Participants

With a main focus on PWLE perspectives, other stakeholders (Caregivers, Care team members) and perspectives from organizations (mental health, health care facilities) were also considered important to include. Five groups of stakeholders were invited: (1) PWLE (i.e., lived experience of mental ill-health), (2) Caregivers (e.g., family members, informal caregivers), (3) Care team members (mental health professionals (e.g., psychiatrists, psychologists, nurses); allied health professionals (e.g., occupational therapists, social workers), somatic/physical health professionals (e.g., general practitioners, public health doctors, preventive medicine experts), (4) Representatives of mental health organizations (non-governmental organisations, professional organisations, people with lived experience), and (5) Managers of health care facilities (managers of (health) organisations), i.e., community health centres, practices, hospitals, preventive centers, social care centers). Included were adults of at least 18 years of age with sufficient knowledge of the local language. The group was determined by self-report of the participants. Semi-structured individual interviews (*N* = 85) were conducted in Austria (*n* = 24), Greece (*n* = 21), Poland (*n* = 20), and Spain (*n* = 20) from June 1 to October 31, 2023.

### 2.3 Data collection and analyses

Recruitment included various channels, such as personal contacts, leaflets, mailing lists, and communication channels of mental health services and NGOs that were contacted by the research team. Existing networks were utilized, and purposive sampling was employed. The sampling framework is inspired by the work of Braun and Clarke (18) on the conceptualization and design for thematic analyses, aiming for a range of participants and oriented on data sufficiency. As such, the analysis followed a reflexive approach also inspired by Braun and Clarke (18). This approach understands themes as the product of interpretive engagement with the data rather than pre-existing categories. The analysis was primarily deductively informed by the study objectives (barriers, facilitators, and diagnostic overshadowing) while remaining open to patterns identified through iterative engagement with the data. In this study, the analysis was theoretically sensitized by concepts of stigma and diagnostic overshadowing. Given the exploratory nature of the objective and the broader cross-country design, we intended to reach a minimum sample size of 70 participants, comprising *n* = 30 PWLE and *n* = 10 from each of the other stakeholder groups, to focus on PWLE experience. We strove for equal distribution across countries and aimed to recruit *n* = 8 PWLE and *n* = 3 participants from each stakeholder group, resulting in a planned sample of 70–80 participants, accounting for potential local differences in reach. People interested in participating in the study contacted the study team directly to schedule an appointment. The semi-structured interviews were conducted in person, via phone, or online and recorded. To ensure participants’ safety, trained professionals (i.e., in health care, social work, and social sciences) conducted the interviews. Participants were informed that they could decline to answer questions and discontinue the interview at any time. Interviews were transcribed verbatim, coded, and thematically analysed afterwards (18). Different individuals conducted the interviews in the respective local language. Initial coding and translation into English were facilitated in each country individually, whereas the development of themes was led by the Austrian partners. As part of this process, the initial codes were grouped and used to identify distinct categories and overarching themes. Taking advantage of the reflexive practice of Braun and Clarke’s (18) approach to thematic analysis is particularly useful in a multi-country, large-scale project setting; especially in the context of a shared meaning-making in a heterogeneous sample with different stakeholders and across countries. To organize the coding processes, regular meetings were held to share and discuss coding procedures and identify potential difficulties. However, given the amount of data and local languages, coder disagreements were discussed and consensus in concepts and theoretical understanding during the coding process was aimed for to ensure high-quality output of the interview data. Still, to investigate the particular context of health care settings while including the views of relevant stakeholders and taking into account the specific phenomenon of DO, a thematic approach according to Braun and Clarke (18) was the appropriate method of choice. The final stage of theme development emphasized the European context across countries while accounting for country-specific differences by iteratively checking for similarities and discrepancies between the four countries, Austria, Greece, Spain, and Poland, and was led by the Austrian partners. Thus, the synthesis of cross-country results was country-descriptive rather than country-comparative. The adequacy of context and interpretation of the results were then revised again by researchers from each country and corrected where necessary. The analysis was deductively driven and oriented toward the study objectives, while it allowed for inductive emergence of themes. While working as a cross-country, multi-disciplinary team of researchers, the authors are aware of the importance of reflexivity in qualitative research and acknowledge their individual professional backgrounds, consisting of medicine (IG, TG, IZ), psychology (CFG, MPG, MK, HMM, JR, KS), health sciences (NMS), nursing (CFG, MPG, NMS), epidemiology / public health (VR, MAW), and social work (AGS, RGT).

## 3 Results

### 3.1 Demographics

A total of 85 individuals across pilot sites in Austria (*n* = 24), Greece (*n* = 21), Poland (*n* = 20), and Spain (*n* = 20) have participated in this qualitative interview study, of whom the majority (*n* = 57) identified as female, with most participants identifying as PWLE (*n* = 36), followed by care team members (*n* = 15), representatives of mental health organizations (*n* = 12), service managers (*n* = 12), and caregivers (*n* = 10). The age ranged from 22 to 76 years (*M* = 47.01, *SD* = 12.95). For an overview, see Table 1.

**Table 1:** Demographics and participant characteristics.

Demographics		Total	Austria	Greece	Spain	Poland
Age in years *M* (*SD*, range)		47.1 (12.95, 22–76)				
Number of participants			24	21	20	20
Gender	Male	28	6	10	3	9
	Female	57	18	11	17	11
Total		85				
Participant characteristics						
PWLE		36	10	10	8	8
Caregivers		10	2	2	3	3
Care team member		15	6	3	3	3
Representatives of mental health organizations		12	3	3	3	3
Representatives of service managers		12	3	3	3	3
Total		85	24	21	20	20

Demographics of participants. PWLE = People with lived experience. M = mean. SD = standard deviation.

The thematic analysis resulted in three themes. The first theme, *Factors located within the health care system*, which refers to factors situated in the social or systemic surroundings of a person, has two categories, namely *Barriers to health care access and utilization* and *Facilitators to health care access and utilization*. The second theme, *Factors in regard to the personal situation*, which refers to factors within the person or related to personal circumstances, has two categories, namely *Hindering factors and behaviors with (potential) negative health consequences* and secondly *Resources and health promoting behavior*. The third theme was *Impact of diagnostic overshadowing on health*. The themes are outlined in more detail below.

### 3.2 Factors located within the health care system: Barriers to health care access and utilization

This theme revolves around system-inherent barriers, which were reported to occur during utilization of services and throughout social interactions within the health care system. The identified barriers included insufficient resources across different areas, such as time, flexibility, financial constraints, and communication issues, with particular country contexts pointed out for each section. Not only has a lack of person-centred, tailored health care been emphasized in this area, but inequities have also been addressed. Across all four countries, experiences of stigma and discrimination within the health care system and not being taken seriously by health care workers (HCW) were repeatedly mentioned.

Considering time constraints as a barrier, waiting times for medical appointments were repeatedly mentioned, especially by participants in Austria, Greece, and Poland. Participants reported little time for HCWs during their visits, potentially impacting the trusting relationships and instead leading to insecurities or even anxiety in patients. Insufficient timely resources were seen as connected to a lack of personnel and low density of medical services, especially in rural areas. It was reported that PWLE may find it challenging to keep appointments at times, and no time is scheduled for additional support, for example, to make appointments or to carry out the given recommendations. Little flexibility to adapt to the individual needs of PWLE was mentioned (e.g., giving the possibility for having consultations with doctors online or via phone, when visiting physically is burdensome) as one participant stressed the necessity of individual needs to be considered:

“*I always tell people, we are different. Because, I think, the basic mistake of the doctor, if a doctor generalizes. We are born as we are individuals; we are individual*.” (PWLE, Poland)

In Austria and Poland, participants noted issues with financial resources, noting differences between public health care and privately paid health care. Having little financial resources was seen as directly impacting the possibility of paying for better quality health services, as one participant stated:

“*(…) when I consult someone privately. They take a lot more time and this gives me much better diagnostic options. You can make a lot of diagnoses not only through - especially in the mental health area, but also in the area of other illnesses - a lot can be made through feedback from the patient. Because I cannot do that without a basis of trust, so that people do not report it back, yes, the diagnosis and treatment can only get worse and not better.*” (PWLE, Austria)

Additionally, providers charging a fee for missed appointments were viewed critically. Not only were financial resources reported to determine the quality of care, but the aforementioned difficulties in meeting appointments could potentially increase the financial burden.

Furthermore, participants in Austria, Greece, Poland, and Spain spoke of a lack of communication, cooperation, and exchange among different professional groups, leading to limited interdisciplinary exchange and sensitization to interactions between mental and physical health. Meanwhile, some participants perceived it as a burden if they were repeatedly referred from one medical doctor to the next. Others described uncertainty about healthcare processes or contact points, as one Polish professional described it:

“*And prevention? Well, it’s also I think at such a basic level. It’s also not like I regularly check where the programs are and what can be done. No. And what’s worse, well I’m a psychiatrist, but what’s worse I think is that patients rarely get this information from other doctors either*.” (Representative of service managers, Poland)

Participants across all four countries repeatedly referred to stigmatization and discrimination of PWLE, and explicitly named as such by some:

“*The stigma surrounding mental illnesses, such as psychosis, can lead to social isolation and even make it difficult for individuals to engage with or seek help from the healthcare system. Healthcare professionals might also exhibit bias or stereotypes, affecting their interactions with these individuals*.” (Representative of Mental Health Organization, Greece)

According to participants, such experiences could lead to feelings of frustration and fear, hindering PWLE from seeking professional help. Participants mentioned experiences and behaviors implicitly referring to stigma and discrimination by HCW, in attitudes and treatment, due to little knowledge about and experience with mental ill-health. Additionally, during interactions with PWLE, a lack of understanding, appreciation, and respect toward PWLE was revealed. Not being taken seriously was a prominent barrier, as participants mentioned that PWLE were instead often viewed as “crazy,” labelled as “hypochondriac,” treated as dumb, accused of being uncooperative, shouted at, being told to “pull themselves” together, or denied access to examination by HCW. The consequences of such experiences might include mistrust, avoidance, and resistance to using the health care system, and turning toward self-treatment instead.

### 3.3 Factors located within the health care system: Facilitators to health care access and utilization

Aside from system-inherent barriers, participants also identified system-inherent facilitators that occur throughout social interactions when using the health care system. The areas mentioned included adaptation to the needs of PWLE, the skills of HCW, the orientation and coordination within the health care system, and health education and literacy. Mutual openness and respect seem to be anchors for PWLE and HCW in creating the best possible health care.

The most prominent facilitator referred to was the adaptation of services to the needs of the individual in the Austrian, Greek, and Polish contexts. This includes methods such as joint decision-making and tailored health care that take into account the patient’s personal context and their preferences. To adapt accordingly, the recognition of PWLE‘s needs was emphasized throughout as a crucial first step. Awareness of HCWs was seen as a necessity; they should consider comorbidities and be open to expanding their knowledge on mental ill-health. However, PWLE would also need to be open about the diagnosis, so that HCW can make changes accordingly (e.g., adjust medication), provide information during the consultation in a different manner (e.g., in written form in case of concentration problems of the patient), and adapt ways of communication (e.g., possibility of answering emails after the consultation in case of questions). One participant stressed the advantage of being reminded of an upcoming appointment:

“*Exactly, and it’s also good practice in some places to call the day before and ask if you can confirm your appointment. I, for example, am very happy when they call me because it reminds me, for example, that I have an appointment tomorrow, oh! I did not put it in my calendar*.” (PWLE, Poland)

According to participants, recognizing PWLE’s needs often means providing a low threshold. The modality of “time” was mentioned as a potential barrier in various ways; here, a low threshold was suggested to be realised with telephone or online consultations, home visits, short-term appointments, or visits without an appointment. Further, the mentioned issues with shame and stigma were suggested to be targeted with discretion regarding the diagnosis.

Common ideas were mentioned across all four countries about the skillset of HCW, who should be interested, empathic, behave respectfully, have sufficient resources of time, and have trust in people with mental health problems. Expanded knowledge, awareness of mental and physical ill-health, and more sensitization were wished for, potentially leading to better experiences in health care settings, as described by one participant:

“*When I was in the emergency room…a resident showed me great kindness and understanding, and spoke to me for a long time and we avoided hospitalization…She gave me time, she gave me space*.” (PWLE, Greece)

Individual needs were emphasized for recognition; for example, it was considered important that HCWs would balance autonomy, self-reliability, and caregiving with PWLE and giving health-related information transparently without causing fear.

According to participants, complex and fragmented health care systems can be perceived as burdensome for coordination and organisation due to increased paperwork and multiple points of contact. A wish for interprofessional cooperation was mentioned in Austria and Spain, for example, to create a network of different professionals. One caregiver mentioned:

*“She [my mother] now actually has a social worker who visits her twice a week. That was the most important thing for me, that she was at least somehow connected to the system again, because when someone from the system is there, the rest is somehow much easier to connect.”* (Caregiver, Austria)

Peer support in the form of EX-IN (experienced involvement) workers, who are specially trained peer workers, within the health care system, could improve the quality of care mentioned in Austria. Support in navigating the health care system was considered helpful, for example, assistance with appointments or with maintaining a healthy lifestyle. There was a clear wish for more participative approaches, so that the voices of PWLE are recognized on a systemic level. In Austria, some participants noted that electronic data recording tools used in the health care system (i.e., ELGA) could simplify collaboration among different professionals. On the other hand, some recommended withdrawing from the data recording as a patient.

Health education was mentioned in Greece, Poland, and Spain as facilitator and health literacy was mentioned in Austria. On a personal level, individuals would be empowered through better health education and health literacy, enabling them to verbalize a problem, ask for help, and demand medical investigation with greater vigor. Furthermore, better health literacy at the societal level could promote health awareness and the importance of preventive measures, while also reducing scepticism against the medical field.

### 3.4 Factors in regard to the personal situation: Hindering factors and behaviors with (potential) negative health consequences

Hindering factors and behavior with (potential) negative health consequences were reported to occur due to living with mental health problems. It includes behaviors with potentially negative health consequences, which are (inter-)personally located, for example, due to prioritizing mental problems, symptoms of such, health care procedures, psychotropic medication, or in daily life. Importantly, it became apparent when and how the living experience of mental ill-health can impact health and health behaviors.

Mental ill-health symptoms, which were mentioned as hindering factors in Austria, Poland, and Spain, include lack of energy or drive, emotion regulation problems, concentration problems, mistrust or paranoia, self-neglect, somatization, and self-harming behavior. Additionally, substance abuse as the cause of health damage and tobacco use as a common risk factor came up in these three countries. Commonly mentioned across all four countries was that such mental health problems are mostly prioritized before other health problems, which may reflect internalized prioritization patterns shaped by prior healthcare experiences:

“*The psychological, psychiatric issues take priority, as it is definite that there is a problem there so I first prioritize my psychiatric issues and then the rest of my health*.” (PWLE, Greece)

Additionally, mental health problems were said to influence multiple steps of a medical consultation, and aversion to going to the doctor’s office can directly impact access to health care, which was especially brought up in Austria and Greece. From difficulties keeping appointments to uneasiness in using public transport, those factors can make it difficult to get to the doctor’s office. Furthermore, crowded or loud waiting rooms can be challenging for some PWLE. During the consultation with the doctor, concentration problems could influence the ability to process and remember the given information:

“*Some people may feel anxious about going alone, not having someone to accompany them, or lacking the social skills to navigate a hospital visit, including waiting in a queue. This is where the need for assistance from the organisation comes in.*” (Care team member, Greece)

Health care procedures and medical examinations could be experienced as frightening and thus be avoided, additionally due to fear of getting a severe illness diagnosed. Preventive measures and information on potential illnesses can lead to feelings of anxiety and are therefore often avoided as well, as the following quote shows:

“*Exactly, because I now have a patient who actually has a (...), something tumour-like on her breast and they have actually told her it’s probably breast cancer and she does not want to undergo treatment, for example.*” (Care team member, Austria)

Participants across all four countries repeatedly referred to psychiatric medication as a hindering factor. Side effects of medication, whether perceived or feared (e.g., weight gain, loss of libido, concentration problems, lack of drive), could influence physical activities, lead to a negative body image due to weight gain, or bad self-perception. One PWLE mentioned changes in physical appearance:

*“Please try to imagine that the side effect on my body was, when I was 16 or 17, due to receiving maximum doses of medication, stretch marks appeared. I cannot do anything about these stretch marks now”* (PWLE, Poland)

Perceived emotional numbness due to medication could lead to self-neglect. Strong night medication could impair physical activities in the evening or the ability to be active the day after. Therefore, a preference for self-treatment in terms of substance or alcohol use was reported by some participants, for example, one participant from Poland stated:

*“Once, I’ll be open about it, when I had a psychological crisis due to poorly prescribed medications, I had hallucinations and delusions. At that time, I simply mixed the medications with alcohol, and it was a deadly combination. As you can imagine, I ended up in the hospital.”* (PWLE, Poland)

Mental health problems were reported to impact health-related behaviors in daily life in different ways. In particular, managing daily life can be challenging, and self-awareness of bodily sensations, physical health, and potential symptoms may be impacted or low. PWLE’s self-perception was characterized by self-blame and feelings of guilt and shame, which were brought up by participants in Austria and Poland, which could further exacerbate self-stigmatization. Additionally, the capacity for social interactions was mentioned in Austria and Greece. PWLE could be socially withdrawn, lack social support, or feel discomfort in group settings or crowded places like hospitals. It was also noted that not all social contacts may be beneficial, and some may influence health behavior negatively.

### 3.5 Factors in regard to the personal situation: Resources and health-promoting behavior

According to participants, living with mental ill-health can be accompanied by many challenges, and every individual situation implies a specific composition of resources as well. Consequently, better and equitable health care may also mean identifying and maintaining those factors. In this regard, several factors related to the personal situation that could be potentially beneficial for health, such as social support, positive emotions, regeneration, and self-awareness, were mentioned by participants. In particular, having access to social support was a major topic. It was seen as beneficial for health in general to participate in social daily life and to have contact with the so-called “world outside”:

“*What do I do? Well, I certainly try to, as I said, as much as possible eat well, as much as possible, take care of physical activity, as much as possible take care of some outings after outside the house, that is, as much as possible be active here at classes. Be active to just, as if to say, have the opportunity, so to speak, to be active in life, outside of work and home*.” (PWLE, Poland)

Social support from friends and family members was mentioned across all countries. Social contacts should be supportive and care about PWLE; for example, by helping them access professional help when needed, supporting them in finding the right specialist, or inspiring them to follow a healthy diet.

“*I have my mother, my father, and sister, who support me tremendously. If it wasn’t for my mother, I may not have asked for help about my physical health*.” (PWLE, Greece)

Additionally, physical health matters of PWLE were considered important in their social network, too. One family member mentioned:

*“Generally, prevention in health matters is important for my son. It is important for all of us, but even more so for my son because he takes a lot of medication.”* (Caregiver, Greece)

Another resource that was emphasized was the importance of having positive experiences. Additionally, positive emotions were seen as a motivational factor in many activities with potential health benefits, such as walking, creative activities, and reading. Other motivational aspects were mentioned in Austria, Greece, and Poland, including self-determination and intrinsic motivation as a necessity. One PWLE recognized the preventive potential of being active:

“*Well, certainly this kind of exercise and physical activity is, in my opinion, such a great preventive measure for almost everything*.” (PWLE, Poland)

In addition to being active, regenerative strategies and rest were considered important. Mindfulness and body awareness were mentioned, for example, by exploring body therapy or acupuncture. One participant mentioned:

“*I practice mindfulness in observing my moods, and when something physically bothers me, I try to work with it using my various somatic methods. Particularly with my stress-related excessive tension, which I think causes the headaches that bother me the most*.” (PWLE, Poland)

Coping strategies were suggested, including skills training, going to psychotherapy, chewing gum instead of smoking, getting sufficient sleep, and eating a healthy diet. Aspects in relation to one’s own self were brought up in Austria, Greece, and Poland. Participants mentioned self-care, empowerment, self-esteem in one’s own ability to distinguish between mental and physical ill-health, resilience, stress reduction and appreciating one’s own needs and boundaries (e.g., going on sick leave if necessary). Self-efficacy was considered important, and appreciation of one’s own improvements was considered a resource for health behavior.

Resources might also overlap and accumulate. One participant from Austria reported that social support from a close person enhanced their self-esteem and had a positive influence on their self-perception regarding mental and physical (ill-) health:

“*And since then, I’ve somehow become more self-confident about it and say: ‘Although I’m mentally ill, that does not mean that I’m not also physically ill*’, *a sentence I now say.*” (PWLE, Austria)

### 3.6 Impact of diagnostic overshadowing on health

Diagnostic overshadowing (DO) refers to a situation in which physical symptoms are attributed to a pre-existing psychiatric diagnosis, thereby delaying or preventing appropriate somatic assessment. In participant’s accounts, such dynamics were described in clinical encounters following disclosure of a mental health diagnosis. In addition to direct instances of DO, participants also reported broader forms of systemic bias associated with mental ill-health. Beyond clinical encounters, participants often described how psychiatric diagnoses could influence their perceived credibility and agency in interaction with institutions. While these experiences do not represent DO in the strict sense, they reflect a broader bias linked to mental health diagnoses that may shape healthcare interactions. For example, being diagnosed with a personality disorder and substance abuse was mentioned to potentially influence a person’s agency in society and the quality of the contact with offices and authorities, or more generally, the systems in which societies are structured. One participant framed it as follows:

“*But with the borderline diagnosis and cannabis abuse that I have in my diagnosis, I never had the feeling that I had a chance. Because I was actually in contact with everyone, whether it was the youth welfare office at the time of the breakup, or I got information from the court - as soon as my diagnosis was revealed, I noticed in people’s eyes how it switched*.” (PWLE, Austria)

“*(…) so, it did not matter what I did, it was a personality disorder and over time I experienced that as very fatal*.” (PWLE, Austria)

In clinical settings, more direct instances of diagnostic overshadowing were described. Some participants described that they had been treated differently by HCW; experiences and examples were repeatedly described as being more difficult after a mental health diagnosis was disclosed. Participants mentioned that if a patient was already in psychiatric treatment, openness for unbiased examination of physical illness was reduced:

“*If a patient is treated with medication for psychological problems, then other problems (symptoms) are explained by the effect of the medication, which may or may not result from this. Or [doctors often say]* e.g.*, “because I change medication, you may experience such symptoms.” - but again: it may or may not result from this.*” (Representative of Mental Health Organizations, Poland)

“*If a patient reports symptoms of concern, very often this is brushed aside, downplayed or left for further observation*.” (Representative of service managers, Poland)

There was the perception that more evidence than usual is needed to be taken seriously for a somatic symptom or a suspected physical illness. Thereby, participants often feel they need to find doctors they can trust and where they can speak openly about their mental health diagnosis, without being reduced to it, which is not always possible in acute care settings:

“*But of course, when something is more acute, I realise that there is a reaction like: ‘Ah! A bipolar disorder’. So, if you understand what I mean*.” (PWLE, Austria)

Diagnostic processes for a physical illness seem to need more proof; for example, in the form of blood tests, as mentioned in Austria, more stamina from the patient’s side, and much more effort made from the professional’s side as well to discover a physical problem beyond the mental one. One mental health professional from Spain described it as follows:

“*Although not all professionals in my field do this, far from it. But well, for this reason that I say that there is sometimes this stigma of whether or not it is a physical symptom, sometimes when I am told something, I do make a mini-form for the family doctor […] and that I recommend that he goes to his doctor because it is the symptoms that worry me. I do not say more, because I think I’m meddling where I’m not wanted, who knows who they are. But I do make it clear that it does not seem to be a symptom of mental health.*” (Care team member, Spain)

PWLE may need to rely on their own initiative and perseverance if their physical health concerns are not taken seriously by their HCWs. One participant described a situation in which a tumor could only be found after a PWLE insisted on examination and got treatment and surgery afterwards:

“*And he stayed there until he was actually taken seriously by another doctor, where they actually discovered a small tumor, but it was quickly operable and that was the end of it, but this not being taken seriously and possibly even having major disadvantages because of it and where a large tumor develops is something that I have heard very often, which (...), yes, which is difficult for sure (...)*.” (Care team member, Austria)

Some participants also described internalized effects of repeated dismissal, including reduced confidence in interpreting bodily sensations. While not DO in itself, such internalized processes may influence health-seeking behavior and contribute to delayed care. Participants reported uncertainty about whether physical sensations were serious and diminished trust in their own bodily perceptions. In some cases, psychotropic medication was described as affecting body awareness, libido, confidence, and self-esteem. These dynamics were said to potentially lead either to neglect of physical symptoms or to heightened anxiety about bodily changes, requiring additional effort to achieve balanced self-care. As one participant reflected:

“*And I’m still working on getting out of this self-stigmatisation myself*.” (PWLE, Austria)

Repeatedly mentioned issues of self-blame, feelings of guilt, and shame regarding mental health problems were also described as issues, as one participant put it:

“*I’m obviously (…) impaired, which is really unpleasant for me, I do not want to walk through the city (cries). I just have to stay in my apartment (cries)*.” (PWLE, Austria)

Asking further about health promotion and prevention, shame and self-perception came up as well, as the same person responded:

“*Yes, well, it’s really my own fault because I do not do anything, nothing and I just want to rot obviously, but […] I’m too lazy to live, I cannot do all this stuff anymore, I do not have the energy for it. I’m sorry.*” (PWLE, Austria)

## 4 Discussion

The aim of this qualitative study was to investigate the barriers and facilitators in access to health care services for PWLE and to explore experiences with DO across four European countries (Austria, Greece, Poland, and Spain). Through semi-structured interviews, we captured perspectives of PWLE and relevant stakeholders, including caregivers, mental health professionals, representatives of mental health organizations, and managers of health care facilities. The analyses were conducted with a country-descriptive rather than country-comparative focus. Despite differences in local healthcare systems, similar topics emerged across participants, suggesting shared patterns in how mental ill-health influences access to somatic healthcare. Although health care systems differ across European countries, structural characteristics, such as high specialization of medical fields (e.g., psychiatry, primary care, or public health), may contribute to fragmented care and limited consideration of patients` overall health needs. Finally, these shared experiences could be viewed as an indicator of a lack of prioritization of public health measures and prevention across systems, although those would help to prevent different diseases at once, as well as support overall health and well-being. The analyses resulted in three themes: (1) *Factors located within the health care system*; (2) *Factors in regard to the personal situation,* capturing dynamic circumstances and behaviors that may influence health positively or negatively; and (3) *Impact of diagnostic overshadowing on health*.

Regarding the first theme, *Factors located within the health care system,* our findings suggested barriers due to a lack of resources on time and flexibility in Austria, Greece, and Poland, financing in Austria and Poland, and communication across all countries. Alarmingly, experiences of stigmatization and discrimination, and not being taken seriously by HCWs, were prominent shared barriers across all four countries as well. Facilitators that could potentially enhance health care were mostly present during social interactions. Adaptation to the needs of PWLE was mentioned in Austria, Greece, and Poland and skilful HCWs were considered a necessity in all four countries to facilitate working together with patients. These findings are in line with a recent publication analysing data from different countries that reported an almost universal stigmatisation experienced by PLWE from society, and that HCW were unfortunately no exception to that (19). Therefore, stigmatization due to mental ill-health, even in the health care setting, does not seem to be a country- or health care system-specific issue, but rather a global one, affecting PWLE worldwide. Considering that health care is a personal and sensitive area of life makes this even more concerning. Being a patient in a health care system in which stigmatization and discrimination occur, which needs to be investigated as a “*fundamental human right to receive unbiased, person-centered, high-quality health care*” (14). The need for trusting relationships in health care is not only preferable, but their absence can lead to life-threatening consequences. Thus, PWLE are at risk for undertreatment of physical health symptoms (20). Further actions are required to provide high-quality health care and reduce risk factors among this population (21). Therefore, such findings should be considered alarming and interpreted as a much-needed call for action.

For the second theme, *Factors in regard to the personal situation,* the prioritization of mental ill-health appeared as the most prominent hindering factor for physical health activities in Austria, Greece, Poland, and Spain. Access to social support seems to be the most prone resource, alongside positive emotions, as well as opportunities for regeneration and self-awareness. Taking a closer look at preventive measures, for example, smoking cessation, differences in medical care for PWLE compared to individuals with no mental health problems are remarkable. While the prevalence of tobacco substance use and nicotine dependency ranges from 33 to 65% in PWLE (22), only 22% of the general population were tobacco smokers in 2022 (23). At the same time, HCWs are less eager to introduce smoking cessation to PWLE than to other patients. Stumbo and colleagues (15) reported in their qualitative study that 88% of clinicians had doubts that PWLE would follow preventive measures, while 82% of PWLE reported that they would try to follow preventive recommendations from their doctor (15). Reasons for this observation may be to not increase the burden on patients or lack of trust in them to actually quit. Meanwhile, in the case of smoking cessation, quitting is more likely if tobacco use is investigated more frequently. In a recent study, frequent screening over the course of five years revealed that cessation measures in women increased from 14% if screened once for tobacco smoking to 42% if screened five or six times by the primary care provider (24). These are strong indicators that the physical health needs of PWLE are often neglected and should gain more attention within the health care system.

Within the third theme, *Impact of diagnostic overshadowing on health,* the effects of stigmatization and discrimination became apparent, ranging from not being taken seriously during social interactions in health care settings to more difficult situations, such as the necessity of personal initiative by PWLE and professionals to ensure proper examination and diagnostics of physical illness. In addition, recognizing and validating one’s own health seems to be questioned due to experiences of DO as well. Importantly, regarding the definitions and understandings of DO, the way participants discussed experiences in the health care system points to what researchers have identified as DO. While the mortality burden is high for mental disorders, mental health problems are rarely the cause of premature death itself (4). Contributors to premature mortality in PWLE are risky health behaviors, lack of prevention, and delays in diagnostics and treatment (2, 6). Liberati and colleagues (2) facilitated a comprehensive systematic review regarding diagnostic overshadowing in PWLE. They differentiated two phenomena: (1) diagnostic inequalities, which refer to population groups and include underdiagnosis, late-stage diagnosis, and route to diagnosis; (2) diagnostic errors, which refer to the individual patient in terms of missed diagnosis, misdiagnosis, and delayed diagnosis. Although DO may not be easily recognized, it seems pervasive in interactions within the health care system. It may even influence self-perception and self-efficacy in health matters for PWLE.

The research outlined before supports the findings of this study regarding the occurrence of diagnostic overshadowing and inequities. Not only did the results show negative experiences of PWLE in health care settings, but it became apparent that the prioritization of mental health problems in PWLE was similar across most participants, regardless of the perspective they were speaking from, whether it was with lived or professional experience. PWLE seem to benefit from additional support regarding physical health and preventive measures. Still, there is a possibility of experiencing self-stigmatization regarding one’s own physical health. At the same time, participants occasionally wished not to reveal the mental health diagnosis in the health care setting. This phenomenon shows similarities to the conceptual framework of the Minority Stress Model (MSM) by Meyer (25). Here, distal stressors, such as discrimination, become internalized and lead to the proximal stress processes. Those can be followed by self-prejudice and concealment, similar to practices of hiding and not disclosing psychiatric diagnoses. Expanding the MSM from gender minorities to other contexts, for example autism, has been shown to be applicable (26). The plain adoption of the original model in another population must be considered with caution (26). Experiences of stigmatization, expectations of rejection, internalized discrimination, and efforts to conceal can create a stressful experience altogether and impact health long-term. Unfortunately, DO is contributing to worse health outcomes and behaviors among PWLE within health care settings, whereas these should rather be settings of support regarding one’s health. The interrelatedness of DO as a negative health care experience and worse health outcomes should be recognized not only as a driver of additional negative emotions, but also frustration and hopelessness (20) in PWLE, potentially leading to resentment. Thus, it becomes a highly relevant problem of quality of care and ultimately, patient safety (14). However, putting DO in context with well-known concepts such as the MSM might guide the direction for further investigation of DO in terms of self-perception, self-awareness, and self-efficacy. Furthermore, the interrelatedness of the structural and, at the same time, individual nature of DO can be explained within the conceptual framework of epistemic injustice. As introduced by Fricker (27), the concept captures the devaluation of someone’s ‘capacity as a knower.’ It can occur as a ‘decreased credibility’ driven by prejudice (i.e., testimonial injustice) as well as an ‘unfair disadvantage’ in sense-making of social experience driven by a lack of collective capacity (i.e., hermeneutical injustice) (27). Being physically ill enhances the vulnerability to epistemic injustice, for example, when the description of the pain of a patient is not taken seriously by HCW (28). Notably, epistemic injustice is also present in mental health services (29). Even if there is a willingness for active patient involvement, its extent remains unknown, or it may still be followed by paternalistic actions of HCW (29). Taken together, PWLE can find themselves in a situation where they face being physically ill in addition to being stigmatized due to the experience of mental ill-health, potentially resulting in the experience of DO. Finally, it needs to be noted that the underlying main issue in this regard appears to be that DO is hard to disclose during clinical practice. DO seems to be some kind bi-directional invisible veil, which is difficult to recognize at all. Therefore, professionals cannot easily see through and PWLE as patients are challenged to find a way out.

The experience of mental ill-health, as well as devaluation as a patient with physical health problems, can occur at the same time and increase the vulnerability of a person. However, considering intersectionality, there are additional dimensions to be considered, such as gender, race, migration status, and socio-economic status that may overlap with mental and physical ill-health. In this context, it is important to note that the study sample was predominantly female, while gender minorities were not represented, as no participant identified as part of a gender minority. Furthermore, the other mentioned aspects were not included in the present study. The mentioned factors potentially impact the experiences of barriers, facilitators, and DO in health care; such interrelations should be investigated further regarding intersectional experiences in health care (30). The study results emphasize the relevance of personal interaction during health care utilization for PWLE, potentially impacting further health care utilization positively or negatively. Furthermore, a wish for the recognition of individual needs and strengths of PWLE was prominent. Thus, our findings inform several potential next steps. Those include education and training regarding stigmatization and DO for HCW. Education about mental ill-health and the promotion of anti-stigma training can contribute to enhanced awareness among HCW for the needs of PWLE. To reduce prejudice, those trainings should focus on enhancing eye-level contact between different stakeholders in health care settings, according to the Intergroup-Contact-Hypothesis (31). Additionally, the founding of programs for person-centered care could support the recognition of the individual needs of PWLE. One promising approach that could be explored is the implementation of patient navigation, which is a service delivery model aimed at facilitating timely access to healthcare within healthcare systems (32–34). Patient navigation as an adaptive and tailored support could be integrated in the country-specific health care systems in order to recognize the needs of PWLE coordinating the health care system. Consequently, more empirical evidence for conceptual frameworks (35) is required to support DO, facilitating transfer from theory to real-world situations and putting potential solutions into practice.

### 4.1 Strengths and limitations

A major strength of this study is the exploratory and qualitative approach, which enabled investigation from different viewpoints, first and foremost that of PWLE but also caregivers and mental health care professionals. Interviews were conducted in person, via phone or video call which exceeds the reach to individuals with limited mobility due to physical or mental health reasons. The analysis was deductively driven regarding barriers and facilitators, which had significant overlap with the interview guidelines and explains the richness in data for the first and second themes. Further, choosing a combined approach and allowing for inductive analyses enabled the generation of the third theme focusing on DO, a widely neglected phenomenon. These results might contribute to a wider spread of knowledge and discussion on DO. Additionally, on a more systemic level, representatives of mental health organisations and mental health care facilities were invited. The recognition of four European countries in this study allows to gain insight in a cross-country manner as well. However, the results are limited as they do not cover all member states of the European Union and do not focus on country comparison. Furthermore, a European PWLE representatives’ organization is part of the CO-CAPTAIN project consortium, and one PWLE’s feedback was incorporated into the interview guidelines. Naturally, participants, especially PWLE, were not interviewed during an acute crisis. Nevertheless, some shared experiences from more acute situations during their lives which had severe impact on their overall health and encounters in the health care system. A limitation of the study is that participants needed to be able to communicate verbally and in the local languages. This limits the range of outreach to a specific group of participants, which excludes for example individuals using sign language or immigrants who don’t speak local languages. Participants were mainly located in urban health care settings or lived near the city, suggesting potential differences in health care access from participants in more rural areas. Another potential limitation to consider is interviewer bias. Interviewers varied across countries and differed in personal characteristics and in country-specific healthcare settings. Hence, cross-country heterogeneity and interviewer positionality may have influenced the results. The researchers were aware of the power dynamics intrinsic to the interviews and made efforts to mitigate power asymmetries between interviewers and participants, which may nevertheless have influenced some responses. Additionally, qualitative studies, such as the present one, generally rely on self-reported experiences of participants, which provide opportunities for insights but also limit the generalizability of the results.

### 4.2 Conclusion

The aim of this study was to exploratively investigate the barriers and facilitators of PWLE in the access to health and preventive care in regard to the phenomenon of DO. Across four European countries, study participants identified individual, social, and systemic factors as both barriers and facilitators. Furthermore, the role of DO was prominent at the systemic level but appeared to perpetuate onto the individual level as well. The results of this study reflect experiences of stigmatization of PWLE in health care settings in Europe, which are alarming and contrary to aiming for the best possible health care for each individual. Although it became clear that HCW should avoid stigmatizing interactions, improvements in health care policy on a systemic level are necessary. Those not only include equity in access to adequate health care but also the minimization of the threat of physical disease. The present study emphasizes the need to take concrete action to reduce DO in health care settings and promote prevention and health care for PWLE. More education and increased awareness of HCW have become essential, as has the implementation of person-centered and tailored health care to provide the best possible care for PWLE. Finally, empowerment and additional efforts regarding health literacy would be beneficial to support PWLE regardless of mental ill-health, as one participant comprehensively put into words: “*Although I’m mentally ill, that does not mean that I’m not also physically ill*.”

## Data Availability

The data that support the findings of this study are available on reasonable request from the corresponding author. The data are not publicly available due to restrictions e.g., their containing information that could compromise the privacy of research participants.
