# Supplementary material for: “Although I’m mentally ill, that doesn’t mean that I’m not also physically ill” – barriers, facilitators and diagnostic overshadowing in healthcare for individuals with lived experience of mental ill-health
Source: Front Public Health. 2026 Mar 31;14:1739409. doi: 10.3389/fpubh.2026.1739409 (PMC13078382; doi:10.3389/fpubh.2026.1739409)
Supplement: Supplementary file 1 [file Data_Sheet_1.docx]

Supplementary Material 1

Interview Guidelines for Semi-Structured individual Interviews

CO-CAPTAIN: Cancer prevention among individuals with mental ill-health: Co-adapting and implementing patient navigation for primary cancer prevention

During the explanation of what the interview is going to be, the patient navigation model should also be explained.

Interviews will be conducted with:

[1.1 Individuals with mental ill-health 2](#_Toc212726746)

[1.2 Caregivers 4](#_Toc212726747)

[1.3 Care team members 6](#_Toc212726748)

[1.4 Representatives of mental health organizations 8](#_Toc212726749)

[1.5 Representatives of service managers 10](#_Toc212726750)

**Funding statement:** CO-CAPTAIN has been funded by the European Union’s Horizon Europe Programme under the Grant Agreement GA101104784. Views and opinions expressed are however those of the authors only and do not necessarily reflect those of the European Union. The European Union cannot be held responsible for them.

## Individuals with mental ill-health

- Sociodemographic data - age, gender, education level, religious/spiritual views, work situation (health insurance?), living situation, mental health issues, co-morbidities
- [Cancer risk factors:] Do you smoke? Do you drink alcohol (how often/much)? Are you physically active? Do you feel you eat healthy? Are you exposed to the sun? /Do you use sun protection?
- How would you describe your physical health? In your opinion, what impact do your mental health issues (possibly name diagnosis directly) have on your physical health?
- Who would you talk to or where would you seek help if you had a physical health problem or concern? Why would you go there?
- To what extent is physical illness prevention relevant to you? Are you currently taking steps to maintain or improve your physical health in the future and if so, what are you doing in this regard? Which preventive examinations/programmes for physical health promotion are you aware of and which ones have you used, or would you use, and why?
- How would you describe your current knowledge/understanding of cancer prevention measures or cancer symptoms?
  - *Are you aware of any risk factors for cancer?*
- Can you recall any particularly positive experiences with health care regarding your physical health since your mental health issues arose? If yes, what was special about the service?
- Can you recall any particularly negative experiences/ obstacles/ problems (e.g., discrimination, stigma) with health care regarding your physical health since your mental health issues arose? If so, what was your experience?
- To what extent do your mental health issues (name diagnosis directly, if applicable) affect your access/use to health services related to your physical health? Can you recall specific barriers you have encountered when seeking support for physical health problems?
  - *Have you ever chosen not to seek support (or delayed seeking support) and if so, what did you base your decision on?*
- Have you ever been offered advice on smoking, your diet, and being physically active (or other forms of cancer prevention), and if so, could you tell me about your experience?
- What do you think would improve physical health care services? How could relevant services be better tailored to the needs of people with mental health issues?
- What do you currently do or would like to do to improve your physical health and well-being?
  - *Direct follow-up question: exercise, improving diet, reducing substance use, mindfulness, meaningful employment.*
- We are working to develop a cancer prevention model for people with mental health issues. Patient navigators (explain what they are: they help persons with health issues to navigate through the healthcare system by e.g. setting up appointments for doctor visits) will offer regular meetings to discuss possible health prevention measures with people with mental health issues and to connect them with prevention measures. What do you think about this idea? Do you think there are certain factors we need to consider?
  - *What could be done to ensure that people with mental health issues receive adequate advice on cancer prevention measures, such as reducing substance use, improving diet, and exercising regularly?*
  - *How do you think we could achieve committed participation of people with mental health issues in cancer prevention?*
  - *What could be done to ensure that people with mental health issues receive access to appropriate cancer prevention measures (e.g., smoking cessation, information on diet and physical exercise)?*
  - *Would you anticipate barriers to the implementation of a better-coordinated model of care that links health and social service providers?*
- At three focus group sessions individuals with mental health issues, caregivers, care team members, representatives of mental health organizations, and representatives of service managers come together in a moderated setting to discuss the issues raised here and to develop the proposed model together. Would it be an option for you to participate in them?
- There is the possibility of collecting data using your mobile phone for another planned part of the study project if you give your consent. This data would be used to improve the navigation process and your quality of life. The data will be treated confidentially only for research purposes.
  - *Would you like to participate in a pilot to check how technology may help in cancer prevention? Could you imagine consenting to data collection of further data that your mobile phone collects (e.g., on movement)?*
- Is there anything else you would like to add to this discussion, or do you have any questions for me?

## Caregivers

*When dealing with family members it should be kept in mind when asking questions that they often do not have any help.*

- Sociodemographic data - age, gender
- Could you start by briefly explaining your role and how often and how you interact with people with mental health issues?
- Are there any physical health issues or needs of the person you care for (name if possible) and if so, what are they?
  - *Which specific physical health problems/complaints (in particular, cancer risk factors) does the individual with mental health issues that you care for have? From your experience, how do these differ from people who do not have mental health issues?*
  - *What impact do you think mental health issues have on physical health?*
- In your opinion, to what extent is physical illness prevention relevant? Which preventive examinations/programmes for physical health promotion are you aware of and which would you recommend to the person you care for (name if possible) and why?
- How would you describe your current knowledge of cancer prevention measures or cancer symptoms?
  - *Are you aware of any risk factors for cancer?*
- What advice would you give to the person you care for if they had a known risk factor for cancer (or other health-related physical health problem)? Where would you suggest the person seek help/support?
- Which barriers/problems, if any, have you experienced or are you aware of when trying to support people with mental health issues to access health care related to physical health - and specifically cancer-related services?
  - *Only if relevant: How easy is it for you to connect the person you care for to relevant physical health services?*
  - *How would you describe the accessibility to relevant services in this area?*
  - *Can you think of any (particularly positive/negative experiences) examples?*
- What do you think would improve physical health care services? How could relevant services be better tailored to the needs of people with mental health issues?
- What do you think people with mental health issues could do to improve their physical health and well-being (e.g., *exercise, improving diet, reducing substance use, mindfulness, meaningful employment)?*
- We are working to develop a model of cancer prevention for people with mental health issues. Patient navigators (explain what they are: they help persons with health issues to navigate through the healthcare system by e.g. setting up appointments for doctor visits) will offer regular meetings to discuss possible health prevention measures with people with mental health issues and to connect them with prevention measures. What do you think about this idea? Do you think there are certain factors we need to consider? For example, in relation to their specific health or support needs?
  - *What could be done to ensure that people with mental health issues receive adequate advice on cancer prevention measures, such as reducing substance use, improving diet, and exercising regularly?*
  - *How do you think we could achieve committed participation of people with mental health issues in cancer prevention?*
  - *What could be done to ensure that people with mental health issues receive access to appropriate cancer prevention measures (e.g., smoking cessation, information on diet and physical exercise)?*
  - *Would you anticipate barriers to the implementation of a better coordinated model of care that links health and social service providers?*
- At three focus group sessions individuals with mental health issues, caregivers, care team members, representatives of mental health organizations, and representatives of service managers come together in a moderated setting to discuss the issues raised here and to develop the proposed model together. Would it be an option for you to participate in them?
- Is there anything else you would like to add to this discussion, or do you have any questions for me?

## Care team members

- Sociodemographic data - age, gender
- Could you start by briefly explaining your role and how often and in which way you interact with people with mental health issues?
- In your experience, what are the typical physical health needs of people with mental health issues?
  - *Which specific physical health problems/complaints (in particular, cancer risk factors) do people with mental health issues tend to have? How do these differ from people who do not have mental health issues?*
  - *What impact do you think mental health issues have on physical health?*
- In your opinion, to what extent is physical illness prevention relevant and beneficial? Which preventive examinations/programmes for physical health promotion are you aware of and which would you recommend to people with mental health issues and why?
- How would you describe your current knowledge of cancer prevention measures or cancer symptoms?
  - *Do you think your knowledge (from your training and current knowledge) is sufficient? Would you like to gain more knowledge on this topic?*
  - *How confident would you be in offering cancer prevention advice/referral to one of your clients?*
- What advice would you give to a person with mental health issues whom you work with if they had a known risk factor for cancer (or other health-related physical health problem)? Where would you suggest the person seek help/support?
  - *Have you encountered or are you aware of any particular barriers to the involvement/care/treatment of people with mental health issues in relation to cancer prevention (e.g., reducing substance use/smoking, improving diet, increasing physical exercise)? (Individual, service, or system related).*
  - *How easy/difficult is it for you to involve/examine/diagnose people with mental health issues?*
  - *How easy do you find it to connect people with mental health issues to relevant services?*
  - *How would you describe the accessibility to and use of relevant services in this area?*
  - *Can you think of any examples?*
- Based on your experience, how would you characterize current models/approaches to cancer prevention for people with mental health issues? Is there anything (services/approaches) that works particularly well/not well?
  - *(What do think are the benefits of these models/approaches for the patients?)*
- We are working to develop a model of cancer prevention for people with mental health issues. Patient navigators (explain what they are: they help persons with health issues to navigate through the healthcare system by e.g. setting up appointments for doctor visits) will offer regular meetings to discuss possible health prevention measures with people with mental health issues and to connect them with prevention measures. What do you think about this idea? Do you think there are certain factors we need to consider? For example, in relation to their specific health or support needs?
  - *What could be done to ensure that people with mental health issues receive adequate advice on cancer prevention measures, such as reducing substance use, improving diet, and exercising regularly?*
  - *How do you think we could achieve committed participation of people with mental health issues in cancer prevention?*
  - *What could be done to ensure that people with mental health issues receive access to appropriate cancer prevention measures (e.g., smoking cessation, information on diet, and physical exercise)?*
  - *Would you anticipate barriers to the implementation of a better-coordinated model of care that links health and social service providers?*
  - *In your opinion, what would be the added value of implementing the patient navigation model for the system or in your workplace?*
- At three focus group sessions individuals with mental health issues, caregivers, care team members, representatives of mental health organizations, and representatives of service managers come together in a moderated setting to discuss the issues raised here and to develop the proposed model together. Would it be an option for you to participate in them?
- There is the possibility of collecting data using a mobile phone for another planned part of the study project if participants give their consent.
  - *The app could give access to real-life data and reports from your patients. What do you think about this?*
- Is there anything else you would like to add to this discussion, or do you have any questions for me?

## Representatives of mental health organizations

- Sociodemographic data - age, gender
- Could you start by briefly explaining your role and how often and in which way you interact with people with mental health problems?
- In your experience, what are the typical physical health needs of people with mental health problems?
  - *Which specific physical health problems/complaints (in particular, cancer risk factors) do people with mental health issues tend to have? How do these differ from people who do not have mental health issues?*
  - *What impact do you think mental health issues has on physical health?*
- In your opinion, to what extent is physical illness prevention relevant? Which preventive examinations/programmes for physical health promotion are you aware of and which would you recommend to people with mental health issues and why?
- How would you describe your current knowledge of cancer prevention measures/cancer symptoms?
  - *How confident would you be in offering cancer prevention advice/referral to one of your clients?*
- What advice would you give to a person with mental health issues whom you work with if they had a known risk factor for cancer (or other health-related physical health problem)? Where would you suggest the person seek help/support?
- What barriers/problems, if any, have you experienced in accessing physical health care – and specifically cancer-related services – for people with mental health issues at the service and system level? What do you think are the barriers/problems in this area?
  - *How would you describe the accessibility to and use of relevant services in this area?*
  - *Can you think of any examples?*
- Which opportunities do you see for people with mental health issues to prevent cancer in mental health organisations? Is there anything that works particularly well/not well?
- Based on your experience, how would you characterize current models/approaches to cancer prevention for people with mental health issues? Is there anything (services/approaches) that works particularly well/not well?
- We are working to develop a model of cancer prevention for people with mental health issues. Patient navigators (explain what they are: they help persons with health issues to navigate through the healthcare system by e.g. setting up appointments for doctor visits) will offer regular meetings to discuss possible health prevention measures with people with mental health issues and to connect them with prevention measures. What do you think about this idea? Do you think there are certain factors we need to consider? For example, in relation to their specific health or support needs?
  - *What could be done to ensure that people with mental health issues receive adequate advice on cancer prevention measures, such as reducing substance use, improving diet, and exercising regularly?*
  - *How do you think we could achieve committed participation of people with mental health problems in cancer prevention?*
  - *What could be done to ensure that people with mental health issues receive access to appropriate cancer prevention measures (e.g., smoking cessation, information on diet, and physical exercise)?*
  - *Would you anticipate barriers to the implementation of a better-coordinated model of care that links health and social service providers?*
  - *In your opinion, what would be the added value of implementing the patient navigation model for the system or in your organisation?*
- At three focus group sessions individuals with mental health issues, caregivers, care team members, representatives of mental health organizations, and representatives of service managers come together in a moderated setting to discuss the issues raised here and to develop the proposed model together. Would it be an option for you to participate in them?
- There is the possibility of collecting data using a mobile phone for another planned part of the study project if participants consent.
  - *How do you think this technology usage could benefit your studies, policies and/or justification of projects submitted to governments?*
  - *Do you think that this technology could contribute to improving the health and well-being of people with mental health issues and if yes, how?*
- Is there anything else you would like to add to this discussion, or do you have any questions for me?

## Representatives of service managers

- Sociodemographic data – age, gender
- Could you start by briefly explaining your role and (if at all) how often and in which way you interact (now or in the past) with people with mental health issues?
- In your experience, what are the typical physical health needs of people with mental health issues?
  - *Which specific physical health problems/complaints (in particular, cancer risk factors) do people with mental health issues tend to have? How do these differ from people who do not have mental health issues?*
  - *What impact do you think mental health issues has on physical health?*
- In your opinion, to what extent is physical illness prevention relevant? Which preventive examinations/programmes for physical health promotion are you aware of and which would you recommend to people with mental health issues and why?
- How would you describe your current knowledge of cancer prevention measures/cancer symptoms? What measures are in place in your organisation to ensure that patients are aware of and implement cancer prevention strategies?
- What advice would you give to a person with mental health issues whom you work with if they had a known risk factor for cancer (or other health-related physical health problem)? Where would you suggest the person seek help/support?
- What barriers/problems, if any, have you experienced in accessing physical health care - and specifically cancer-related services - for people with mental health issues at the service and system level? What do you think are the barriers/problems in this area?
  - *How would you describe the accessibility to and use of relevant services in this area?*
  - *Can you think of any examples?*
  - *What can organisations do to increase accessibility for people with mental health issues? What can (your) organisations do to remove barriers?*
- Which opportunities do you see for people with mental health issues to prevent cancer in mental health organisations? Is there anything that works particularly well/not well?
- Based on your experience, how would you characterize current models/approaches to cancer prevention for people with mental health issues? Is there anything (services/approaches) that works particularly well/not well?
- We are working to develop a model of cancer prevention for people with mental health issues. Patient navigators (explain what they are: they help persons with health issues to navigate through the healthcare system by e.g. setting up appointments for doctor visits) will offer regular meetings to discuss possible health prevention measures with people with mental health issues and to connect them with prevention measures. What do you think about this idea? Do you think there are certain factors we need to consider? For example, in relation to their specific health or support needs?
  - *What could be done to ensure that people with mental health issues receive adequate advice on cancer prevention measures, such as reducing substance use, improving diet, and exercising regularly?*
  - *How do you think we could achieve committed participation of people with mental health issues in cancer prevention?*
  - *What could be done to ensure that people with mental health issues receive access to appropriate cancer prevention measures (e.g., smoking cessation, information on diet, and physical exercise)?*
  - *Would you anticipate barriers to the implementation of a better-coordinated model of care that links health and social service providers?*
  - *In your opinion, what would be the added value of implementing the patient navigation model for the system or for the service you represent?*
- At three focus group sessions individuals with mental health issues, caregivers, care team members, representatives of mental health organizations, and representatives of service managers come together in a moderated setting to discuss the issues raised here and to develop the proposed model together. Would it be an option for you to participate in them?
- There is the possibility of collecting data using a mobile phone for another planned part of the study project if participants give their consent.
  - *Do you think it could be useful to aggregate this data within the Electronic Health Records and/or in a dashboard to manage your service? Do you see it feasible to implement this tool in your organisation?*
  - *What barriers or difficulties do you think there would be (if any) in the system or on the part of professionals or patients?*
- Is there anything else you would like to add to this discussion, or do you have any questions for me?
